# Focus-RCNet: a lightweight recyclable waste classification algorithm based on focus and knowledge distillation

**DOI:** 10.1186/s42492-023-00146-3

**Published:** 2023-10-11

**Authors:** Dashun Zheng, Rongsheng Wang, Yaofei Duan, Patrick Cheong-Iao Pang, Tao Tan

**Affiliations:** Faculty of Applied Sciences, Macao Polytechnic University, Rua de Luís Gonzaga Gomes, Macao, 999078 China

**Keywords:** Waste recycling, Waste classification, Knowledge distillation, Lightweight, Attention

## Abstract

Waste pollution is a significant environmental problem worldwide. With the continuous improvement in the living standards of the population and increasing richness of the consumption structure, the amount of domestic waste generated has increased dramatically, and there is an urgent need for further treatment. The rapid development of artificial intelligence has provided an effective solution for automated waste classification. However, the high computational power and complexity of algorithms make convolutional neural networks unsuitable for real-time embedded applications. In this paper, we propose a lightweight network architecture called Focus-RCNet, designed with reference to the sandglass structure of MobileNetV2, which uses deeply separable convolution to extract features from images. The Focus module is introduced to the field of recyclable waste image classification to reduce the dimensionality of features while retaining relevant information. To make the model focus more on waste image features while keeping the number of parameters small, we introduce the SimAM attention mechanism. In addition, knowledge distillation was used to further compress the number of parameters in the model. By training and testing on the TrashNet dataset, the Focus-RCNet model not only achieved an accuracy of 92$\%$ but also showed high deployment mobility.

## Introduction

As the living standards of residents continue to improve and the consumption structure becomes richer, the amount of domestic waste generated has dramatically increased. According to the latest report by the United Press International, the amount of global waste will increase by 70$\%$ by 2050 [1]. Environmental problems caused by large amounts of waste are becoming increasingly serious, and the development of waste treatment is urgently required. Waste disposal has a direct or indirect impact on human life and the environment, and classifying waste into different categories based on its nature is a key activity in waste management.

A proper waste management system can treat different types of waste accordingly (e.g., composting, incineration, landfilling, and recycling) and help mitigate the adverse effects of waste. Waste management involves several activities, such as waste collection, classification, and disposal or recycling. The World Bank states that only 13.5$\%$ of global waste is recycled, while approximately 33$\%$ of waste is publicly discarded without any initial classification [2]. This results in different types of waste are freely scattered across a wide variety of environments. To control the environmental impact of waste, waste classification is considered an effective way to improve resource efficiency and protect the environment and has been actively promoted widely as a management measure. However, the implementation of waste separation is problematic due to the wide variety of waste types, low awareness of waste separation among residents, and imperfections in related policies. Currently, waste separation requires considerable manpower for manual classification, which is time-consuming and inefficient. To prevent further environmental pollution and improve the efficiency of waste classification, it is of great academic value and practical significance to study an effective automatic waste classification method.

The development of artificial intelligence has provided new solutions to this problem. With the rapid development of science and technology, especially computer and sensor technology, there have been many improvements and developments in traditional municipal waste management systems [1]. Many scholars have designed smart waste classification algorithms based on deep learning techniques [3–5] that can be directly applied to smart waste classification devices, such as smart bins, waste classification machines, and smart dumpsters. These studies have shown that deep learning applications can accelerate waste classification and detection and effectively improve waste classification efficiency. However, these algorithms suffer from complex model structures, long inference times, and high computational costs. These problems limit the widespread implementation of intelligent waste classification systems in IoT hardware, and the research direction has shifted back to lightweight deep learning models [4, 6]. For complex deep learning models, lightweightness can shorten the inference time and reduce the computational cost, thus adapting to the needs of most IoT devices. However, lightweight models are often accompanied by a decrease in model accuracy. Improving the computational speed of a model while maintaining its high accuracy is attracting increasing attention.

Given this background, this study makes the following main contributions:

1. Our goal is to reduce the dimensionality of the features and retain effective information while avoiding overfitting and loss of information. We apply the Focus module to waste classification for the first time and show its satisfactory results in waste image classification tasks.
2. Models with large computational and parametric quantities are difficult to deploy in certain settings. To address this disadvantage, we adopt a lightweight idea to design the network, which can maintain the characteristics of a larger model with high efficiency and high accuracy while keeping the computational cost and number of parameters small.
3. We aim to make the model focus more on waste image features while ensuring a small number of parameters. Therefore, the SimAM attention mechanism is introduced, and we demonstrate that it can focus on image features efficiently and improve the model accuracy with a small number of parameters.

## Related work

In urban waste management, waste separation and recycling play crucial roles in improving the overall living environment of city residents [7]. Waste classification requires a large amount of human resources and has high cost. Therefore, several researchers have studied waste classification, mainly using traditional methods. For example, Riba et al. [8] proposed a method for detecting and classifying the components of automated waste classification machines. Gundupalli et al. [9] used a thermography-based technique to classify the metallic and non-metallic fractions of e-waste. Bonifazi et al. [10] used an innovative hierarchical classification strategy based on hyperspectral imaging to classify different polymer flakes in mixed plastic waste. Xiao et al. [11] proposed a complementary troubleshooting method for online identification of construction waste, which was used to improve the utilization of construction waste. However, these methods involve complex algorithmic processes and have low recognition rates.

With the rise of deep learning techniques, many effective visual representations and recognition techniques have emerged, which hold promise for designing more effective algorithms for waste classification tasks. Yang and Thung [12] collected 2527 waste images as a dataset called TrashNet. They used a support vector machine (SVM) on scale-invariant features learned by ResNet50 for classification and achieved good results. Similarly, Adedeji and Wang [13] presented a feature encoder that uses ResNet50 as a pre-trained model to extract waste images and an SVM to classify different types of waste. Nowakowski and Pamuła [14] attempted to quickly detect the class and size of e-waste devices in images using a region-based convolutional neural network (CNN). Liang and Gu [15] proposed a multi-task learning architecture based on CNNs, which can be used to simultaneously recognize and localize garbage in images. Zhang et al. [16] proposed the DenseNet169 spam image classification model based on migration learning. Bircanoğlu et al. [4] proposed the RecycleNet model, which reduced the number of parameters in a 121-layer network to three million; however, the accuracy of the model test was only 81%.

Although the use of CNN-based algorithms has led to some improvements in waste classification, the classification accuracy and efficiency of the model require further improvement. The concept of knowledge distillation was first introduced by Hinton et al. [17]. Knowledge distillation focuses on migrating the knowledge contained in model parameters to a new parametric model that aids in the training and classification of other tasks in a limited manner during training [18, 19].

To address the above issues, we designed a lightweight network. We applied the Focus module to the image classification task using the SimAM parameter-free attention mechanism, and we used knowledge distillation to effectively migrate the knowledge learned by the model to the waste classification task. In this paper, we propose a lightweight improved waste classification model that maintains a recognition accuracy of 92% while focusing on implementing a lightweight algorithm.

## Methods

**Fig. 1:**
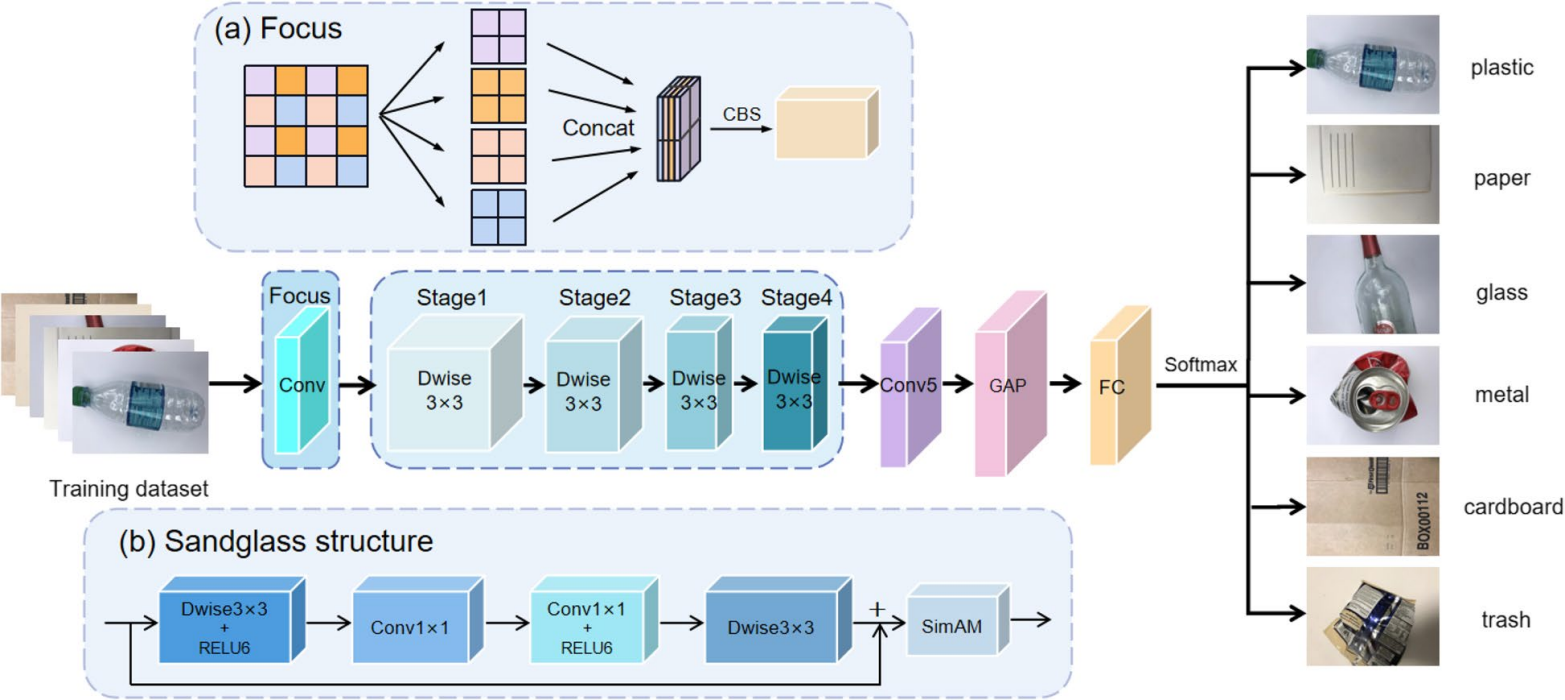
Overall architecture diagram: **a** Focus module; **b** Structure in each layer from the stage

In this paper, we propose a lightweight waste classification model called Focus-RCNet, which can be deployed in mobile terminals. Figure 1 and Table 1 describe the model architecture.

**Table 1:** Description of the architecture of Focus-RCNet

Layer	Output_size	Ksize	Stride	Repeat	Output_channel
Image	380 × 380				3
Focus	190 × 190	1 × 1	1	1	24
Stage1	95 × 95		2	1	48
	95 × 95		1	3	
Stage2	48 × 48		2	1	96
	48 × 48		1	2	
Stage3	24 × 24		2	1	192
	24 × 24		1	1	
Stage4	12 × 12		2	1	384
	12 × 12		1	1	
Conv5	12 × 12	1 × 1	1	1	512
GAP	1 × 1	7 × 7			
FC					1000
Flops					418.8 M

### Focus module

Before the image enters the network layer, this study aims to shrink the image to reduce the dimensionality of the features and retain the valid information to some extent to avoid overfitting without losing information. Common downsampling methods sacrifice some information in exchange for a reduction in data volume.

As shown in Fig. 1 (a), this research model introduces the Focus module in the YoloV5 [20] framework, which aims to reduce the numbers of layers, parameters, and flops and increase the forward and backward speed while minimizing the impact of mean average precision. The Focus process is to first perform a slicing operation on a 224$\times$224$\times$3 image with inter-column sampling, and four independent feature maps are taken and stacked on the image at the same time; at this time the number of channels is expanded to 12 compared with the original three RGB color channels, and the channel dimension is expanded 4-fold. A feature map of 112$\times$112$\times$12 is obtained, and then the feature map is convolved into the SiLU function output, that is, the CBS operation; finally, the 2-fold downsampled feature map with no lost information is obtained.

The Focus layer converts the information in the w-h plane to the channel dimension and then extracts different features using CBS. This approach can reduce the information loss caused by downsampling, thus achieving our ultimate goal.

### Sandglass structure and SimAM attention module

In recent years, bottleneck structures, inverted residual structures, and the sandglass structure used in this study have emerged to construct lightweight backbone networks. It has been experimentally proven that adding deep convolution to the ends of the residual path increases its spatial expressiveness.

In this study, we aimed to design a network that maintains an efficient and highly accurate network structure while also maintaining a low computational cost and parametric volume. Therefore, in this study, the structure was designed by referring to the inverted sandglass structure of MobileNetV2 [21]. This study used depth-separable convolution to perform feature extraction operations on images. Specifically, the feature maps after Focus extraction are first convolved channel-by-channel and then point-by-point. The designed network goes through Dwise3 × 3 convolution and then into two 1 × 1 convolutions, before finally going through Dwise3 × 3 convolution again to output the features shown in Fig. 1(b).

**Fig. 2:**
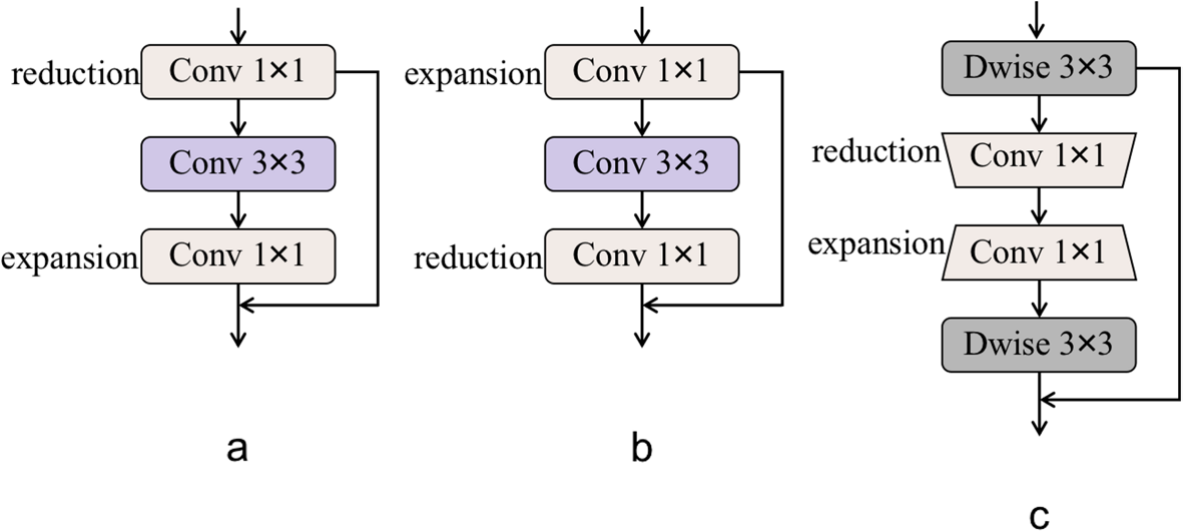
**a** Bottleneck module; **b** Inverted residual block; **c** Sandglass structure

As shown in Fig. 2(a), the bottleneck structure first reduces the dimensionality to reduce the number of channels, uses normal convolution for feature extraction, and finally boosts the dimensionality again. The bottleneck structure not only reduces computational effort but also increases the number of network layers to facilitate training. As shown in Fig. 2(b), an inverted residual structure was proposed for MoblieNet V2, which improves the performance of the mobile network in multi-type task classification. The inverted residual structure first uses 1$\times$1 convolution for dimension generation to obtain more image features, followed by feature extraction using a 3$\times$3 convolution kernel, and finally a 1$\times$1 convolution kernel for dimensionality reduction. However, the reduced feature dimensionality tends to lead to gradient confusion in propagation, which reduces the ability of gradient propagation across layers and thus affects the convergence and model performance during training. Therefore, a sandglass structure is formed. As shown in Fig. 2(c), compared with the inverted residual structure, the sandglass structure creates jump connections between linear high dimensions, can transmit more information in the network structure, and applies deep convolution to the high-dimensional space to learn more expressive features.

As shown in Fig. 1(b), after each stage, our work introduces a general parameter-free attention mechanism, namely, the SimAM [22] attention mechanism. This study is designed to make the model of focus plays more attention to waste image features, while ensuring that the number of parameters is computationally small. SimAM can derive a fast analytical solution to the energy function while discovering the importance of each neuron.The SimAM attention mechanism differs from the traditional 1-D and 2-D attention weights that would limit the ability to learn more discriminative retrieval, and it hopes to pay attention from 3-D attention weights to each neuron in the channel. Yang and Thung [12] argue that the computation of the 3-D weights should be straightforward while allowing the module to maintain a lightweight property by defining the following energy function for each neuron, as shown in Eq. 1.

1 $$\begin{aligned} e_{t}\left( w_{t}, b_{t}, \textbf{y}, x_{i}\right) =\left( y_{t}-\hat{t}\right) ^{2}+\frac{1}{M-1} \sum _{i=1}^{M-1}\left( y_{o}-\hat{x}_{i}\right) ^{2} \end{aligned}$$

where $\hat{t}=w_{t} t+b_{t}$ and $\hat{x}_{i}=w_{t} x_{i}+b_{t}$ are linear transforms of *t* and ${x}_{i}$, where *t* and ${x}_{i}$ are the target neuronand other neurons in a single channel of the input feature $\textbf{X} \in \mathbb {R}^{C \times H \times W}$. $M = H \times W$ denotes the number of neurons in the channel. ${w}_{t}$ and ${b}_{t}$ are the weights and biases of the transformation, respectively.

A network with the stacking structure designed in this study can help the model learn features better and ensure that it has the advantages of a low number of parameters, fast computation, and no loss of accuracy.

### Knowledge distillation and training strategies

This study integrated knowledge distillation into the field of waste classification. Hinton et al. [17] first introduced the concept of knowledge distillation, hoping to achieve knowledge migration using a complex but prediction-accurate teacher network to predict a soft target, and then feed it to a lightweight student network that is more suitable for inference deployment. The knowledge distillation operation has the advantages of accelerating model training, improving performance, and migrating learning.

**Fig. 3:**
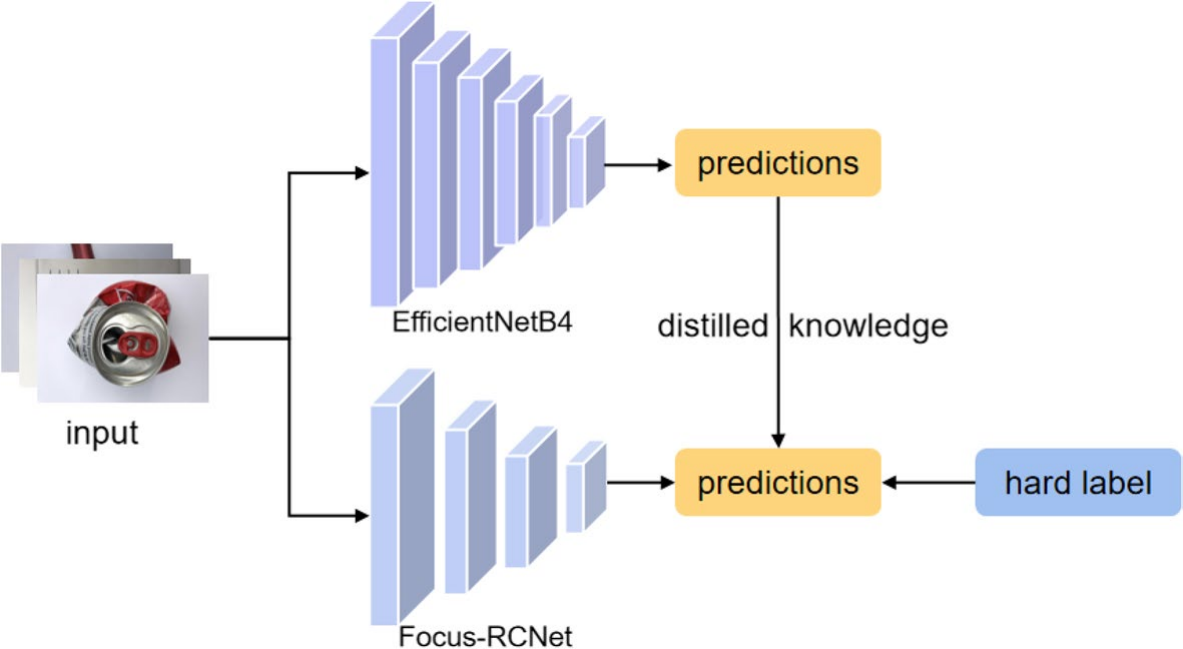
Knowledge distillation architecture

Our study uses EfficientNetB4 [23] as the teacher model, and EfficientNetB4 distillation can help the model obtain high-quality features from the pretrained model. The accuracy of the model designed in this study was 90$\%$, and the accuracy reached 92$\%$ after using EfficienetNetb4 knowledge distillation, which can improve the performance of the network by 2$\%$ compared to the original design. As shown in Fig. 3, the output of softmax using the EfficientNetB4 teacher network yields a soft target with Eq. 2.

2 $$\begin{aligned} q_{i}=\frac{\exp \left( z_{i} / T\right) }{\Sigma _{j} \exp \left( z_{j} / T\right) } \end{aligned}$$

where *T* denotes the temperature. The original softmax function is a special case in which *T* = 1. The higher *T* is, the smoother the output probability distribution of softmax tends to be. The greater the entropy of its distribution, the more information carried by the negative labels will be relatively amplified, and the model training will focus more on the negative labels.

In the teacher network predicted results and in the student network predicted results to calculate loss-soft, while the student network directly predicted results with the real label to calculate loss-hard, total-loss is the combination of both, and the final loss formula is

3 $$\begin{aligned} L=a L^{(s o f t)}+(1-a) L^{(h a r d)}. \end{aligned}$$

where *L* is the total-loss, $L^{(s o f t)}$ is the soft label predicted by the teacher, and $L^{(h a r d)}$ is the hard-loss experienced by students.

These below three subsections first describe the environment and parameter settings used in the model training and the dataset used and then evaluate the model using the classification model evaluation metrics.

### Experimental setup

For training, PyTorch was used to implement the model. This model was trained on the NVIDIA GeForce RTX 3090 Ti server configuration. The algorithm was trained on a 64-bit Ubuntu 22.04 operating system. The parameters were optimized using stochastic gradient descent with a momentum $\beta$ of 0.9, batch size set to 16, learning rate initialized to 0.05, learning rate reduced by a factor of 10 every 90 cycles, and weight decayed to 10-4. All models were trained for 200 epochs. At the same time, cosine annealing [24] learning rate was used in the training to ensure that the model went beyond local optima to the full optimum.

### Datasets and data processing

The dataset used in this study was TrashNet [12], which is a dataset for the classification of waste images. As shown in Table 2, the TrashNet dataset has a total of 2528 images, which are divided into six categories: 594 images of paper, 501 images of glass, 483 images of plastic, 410 images of metal, 403 images of cardboard, and 137 images of trash. This study divided the dataset into 70$\%$ for training and 30$\%$ for validation. In the data processing stage, data enhancement operations were performed on the data. The data augmentation operations used were as follows: (1) random flip and horizontal flip operations, (2) RandomBrightnessContrast, which randomly changes the brightness and contrast of the input images, and (3) cutout, which randomly cuts out some areas of the sample and fills them with zero pixel values. The classification results remained unchanged. Then, the size of the image was converted to 224 $\times$ 224 and normalized. (4) Finally, the image was cropped to a 380 $\times$ 380 pixel RGB image and normalized.

**Table 2:** Experimental dataset information

Classes	Number	Train set (70%)	Valid set (30%)
Paper	594	415	179
Glass	501	350	151
Plastic	483	338	145
Metal	410	287	123
Cardboard	403	282	121
Trash	137	96	41
Sum	2528	1768	760

### Experimental indicators

After the model was constructed, it was evaluated using several performance metrics, including accuracy, recall, and F1 score. This subsection evaluates the proposed waste classification model using confusion matrix, receiver operating characteristic (ROC) curve, area under the curve (AUC), loss value, and accuracy metrics. These evaluation metrics are calculated as follows:

Recall rate indicates the proportion of all matched positive cases, calculated as

4 $$\begin{aligned} \text {Recall }=\frac{T P}{(T P+F N)} \end{aligned}$$

Precision indicates the number of waste samples that predicted TP as positive during waste classification, calculated as

5 $$\begin{aligned} \text {Precision} = \frac{T P}{(T P+F P)} \end{aligned}$$

Accuracy represents the proportion of the type of waste that is correctly classified in the total waste classification, calculated as

6 $$\begin{aligned} \text {Accuracy} = \frac{T P + T N}{(T P+F P+T N+F N)} \end{aligned}$$

F1-score is a judgment index that integrates the two indicators of precision and recall, calculated as

7 $$\begin{aligned} F 1-\text {score} = \frac{2(\text {Recall} \times \text {Precision})}{(\text {Recall}+ \text {Precision})} \end{aligned}$$

This study aimed to comprehensively evaluate the precision and recall of confusion matrices. The method calculates the precision and recall and then calculates an average over each confusion matrix to obtain the “macro-precision”, “macro-recall”, and corresponding “macro-F1”. This is calculated as follows:

8 $$\begin{aligned} \text {macro}-\textrm{F}1&= \frac{2 \times \text{ macroP } \times \text{ macroR } }{ \text{ macroP } + \text{ macroR } }\nonumber \\ \text {macroP}&=\frac{1}{n} \sum _{i=1}^{n} P_{i}\nonumber \\ \text {macroR}&=\frac{1}{n} \sum _{i=1}^{n} R_{i} \end{aligned}$$

## Results

### Confusion matrix

**Fig. 4:**
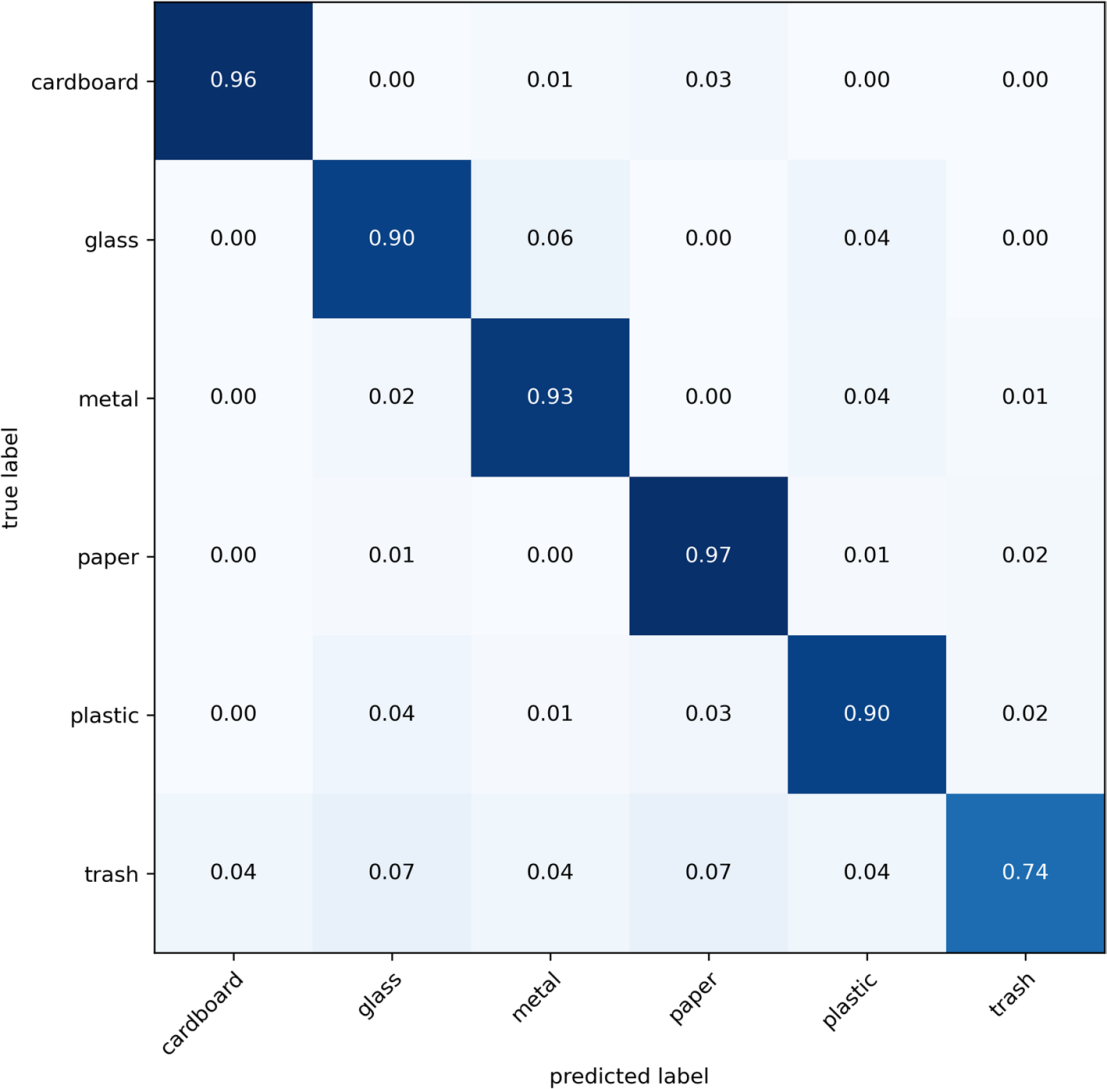
Confusion matrix

The model designed in this study after knowledge distillation was tested using the TrashNet dataset. The accuracy of the model was 92$\%$. The confusion matrix is presented in Fig. 4. Each row of the matrix gives the values predicted for cardboard, glass, metal, paper, plastic, and waste, and each column of the confusion matrix gives the true value of the TrashNet dataset. The diagonal lines of the matrix indicate the images in the correct category, while the values outside the diagonal line indicate the number of incorrectly predicted images. As show in Fig. 4, the accuracy of the proposed model was 96$\%$ for cardboard, 90$\%$ for glass, 93$\%$ for metal, 97$\%$ for paper, and 90$\%$ for plastic. However, it was only 74$\%$ for trash, mainly because the images in the trash image dataset contain other categories of trash, such as paper, plastic, and metal; these are not other waste and therefore affect the overall accuracy of the model in classifying this type of waste. It is also not possible to exclude the fact that this part of the classification is smaller than the data of other classes. The specific data are listed in Table 2.

### ROC curves

**Fig. 5:**
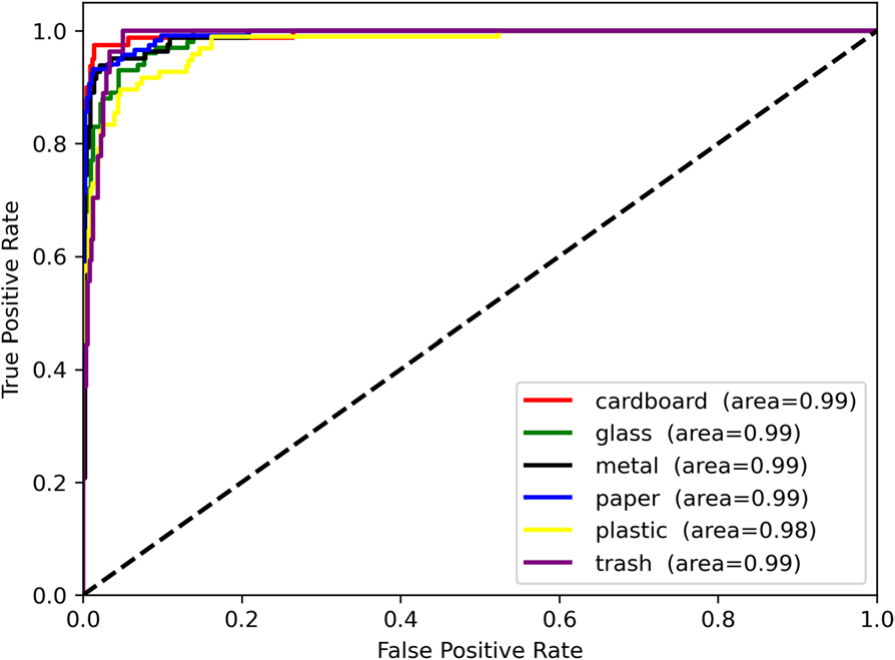
ROC curve after prediction of Focus-RCNet classification model

The ROC curves for the different types of waste are shown in Fig. 5. Waste data for cardboard, glass, metal, paper, plastic, and trash are shown separately in this figure. These six types of samples have similar AUC, similar classifications, and relatively similar accuracy, as shown in Table 3. Therefore, the proposed model works very well.

**Table 3:** Accuracy for each type of waste prediction

Classes	Precision	Recall	F1-score
Cardboard	0.99	0.96	0.97
Glass	0.91	0.90	0.90
Metal	0.89	0.93	0.91
Paper	0.94	0.97	0.95
Plastic	0.91	0.90	0.90
Trash	0.80	0.74	0.77
Macro average	0.91	0.90	0.90

### Ablation experiments

Ablation experiments were conducted to demonstrate the effectiveness of the proposed method. The accuracy of the baseline model used in this study was 88.07$\%$, which increased by 3.13$\%$ with the addition of the Focus module. The SimAM module was then added, and the accuracy was increased by 1$\%$. These experiments proved that all proposed methods were effective. The results are listed in Table 4.

**Table 4:** Results of ablation experiments

Model	Factor		Accuracy (%)
	Focus	SimAM	
Focus-RCNet	-	-	88.07
	✓	-	91.20
	✓	✓	92.20

### Model performance comparison

**Fig. 6:**
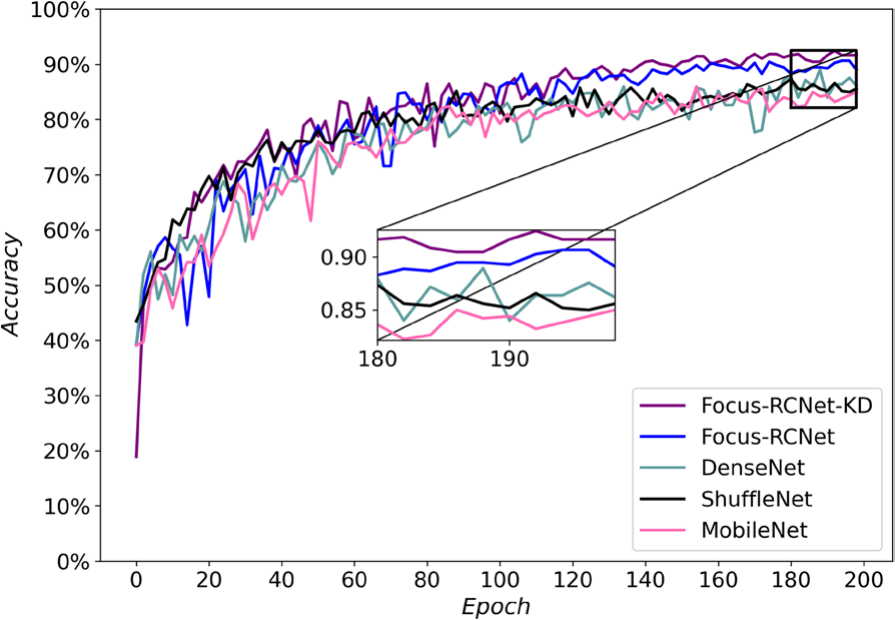
Accuracy of proposed model compared to other networks

Finally, the proposed model was compared with ShuffleNet, MobileNet, and DenseNet on the same dataset, and the results are shown in Fig. 6.

It can be seen from Fig. 6 that the accuracy of the proposed model was 92$\%$ after knowledge distillation and 90$\%$ for the original model, while it was 86$\%$ for ShuffleNetV1, 88$\%$ for DenseNet121, and 85$\%$ for MobileNetV1.

This study also compared some classical large models, and the proposed model showed superior results in terms of accuracy, number of parameters, and computational volume.

In addition, we compared the parametric quantities of Focus-RCNet with those of the teacher model EfficientB4 on the TrashNet dataset. As shown in Table 5, EfficientB4 has 17.559M parameters and 4.49G Flops, while Focus-RCNet has 525.802k parameters and 418.8M Flops. Therefore, the proposed model not only has good performance in terms of accuracy but also has very small parameter number and Flops, as well as high deployability on various devices.

**Table 5:** Model effect comparison

Model	Accuracy (%)	Flops (G)	Params (M)
ResNet50	0.87	12.087	25.560
DenseNet121	0.88	8.121	6.960
ShuffleNetV1	0.86	0.127	0.348
MobileNetV1	0.85	0.952	2.232
EfficientB4(teacher)	0.97	4.490	17.559
Focus-RCNet	0.90	0.418	0.525
Focus-RCNet-KD	0.92	0.418	0.525

## Discussion

We proposed a lightweight CNN model called Focus-RCNet for automatic garbage classification. Compared with traditional CNNs, this model offers higher mobility and smaller computational complexity while maintaining high accuracy. This study addressed the problem of high computational complexity and the complexity of CNNs in practical applications and achieved satisfactory results in garbage classification. However, there are still some limitations to this work. The experiments in this study were only conducted on the TrashNet dataset, and their applicability to other datasets must be further verified. In addition, although the proposed model has high accuracy, it may lead to misjudgments in certain marginal cases. Therefore, we need to explore the limitations and directions for improving the model in future research. Furthermore, we should compare the proposed model with other garbage classification models and explore the advantages and disadvantages of different models. Finally, we must pay attention to the advantages and limitations of this study. The model presented in this paper is considered a good candidate for garbage classification; however, its application in other fields requires further exploration. This paper proposed a lightweight CNN model but ensuring low computational complexity may affect the accuracy of the model, which is a long-standing problem in balancing complexity and accuracy.

## Conclusions

This paper proposed a lightweight network architecture using knowledge distillation to further compress and optimize the model and validate the performance of Focus-RCNet on the TrashNet dataset. The model has the advantages of low computational cost, small number of parameters, high speed, and high accuracy, and it can be well deployed on mobile devices. The results of this study can be used for the automatic classification of waste, which can effectively reduce human intervention. Finally, the model was tested using the TrashNet dataset, and the accuracy of the model reached 92$\%$.

## Data Availability

The public datasets used in this thesis are open access and the data source is stored at https://github.com/garythung/trashnet. We confirm that the data were used and interpreted correctly and we performed data augmentation to enlarge dataset. Detailed information on the dataset and how to use it can be found on the repository of the dataset. We hereby declare that the data used in this study are publicly available and that other researchers are free to access and use them further.

## Abbreviations

SVM: Support vector machine
CNN: Convolutional neural network
ROC: Receiver operating characteristic
AUC: Area under the curve
